# Sexual exploitation of unaccompanied migrant and refugee boys in Greece: Approaches to prevention

**DOI:** 10.1371/journal.pmed.1002438

**Published:** 2017-11-22

**Authors:** Julie Freccero, Dan Biswas, Audrey Whiting, Khaled Alrabe, Kim Thuy Seelinger

**Affiliations:** 1 Human Rights Center, School of Law, University of California, Berkeley, Berkeley, California, United States of America; 2 Faros, Athens, Greece; London School of Hygiene and Tropical Medicine, London, England

## Abstract

In this essay, Julie Freccero and colleagues discuss resources to prevent the sexual exploitation of unaccompanied and separated refugee boys in Greece.

**Summary points**

- The refugee and migrant crisis in Europe has drawn international attention to the issue of sexual exploitation of unaccompanied and separated refugee boys, requiring humanitarian actors and service providers to quickly develop responses in the absence of an established evidence base.
- Although adolescent boys comprise a substantial majority of the population of unaccompanied and separated children, they are rarely the focus of policy discussions and are consistently left out of gender-based violence prevention and response efforts. Gender-specific research, policy guidance, and evidence of best practices related to interventions preventing the sexual exploitation of boys are extremely limited.
- Three prevention approaches have been heavily debated in Greece among policy makers and practitioners: high-security shelter models, life skills education, and cash transfer programming. While lessons can be drawn from evidence of these interventions in other contexts or among other target populations, research on the impact of these approaches on vulnerability to sexual exploitation among unaccompanied refugee and migrant boys is urgently needed to inform policy and program design.
- A combination of approaches, addressing risk factors at multiple levels, such as building individual-level knowledge and skills, providing community- or family-level protection in the absence of traditional support mechanisms, and structural interventions to address economic vulnerability, is likely needed in order to significantly reduce the vulnerability of unaccompanied and separated boys to sexual exploitation.
- Rigorous evaluation of current pilot approaches is critical to building the gendered evidence base, guidance, and resources practitioners urgently require.

## Introduction

Europe’s refugee and migrant crisis is increasingly defined by the unprecedented number of children crossing the Mediterranean Sea, many of whom are unaccompanied and separated children (UASCs) as defined by the United Nations High Commissioner for Refugees and the Committee on the Rights of the Child [1]. UASCs have been separated from both parents and their relatives and are not being cared for by an adult. In 2015 alone, almost 90,000 unaccompanied children applied for asylum in the European Union, approximately 4 times the number of those who applied during the previous year [2]. In 2016, over 63,000 UASCs applied, 89% of whom were males [3]. In Greece in particular, large numbers of migrant and refugee unaccompanied children, the majority of whom are boys between ages 14 and 17, have been stranded without access to adequate accommodation and income-generating opportunities as they await asylum decisions and processing [4]. Over half of the approximately 2,500 registered UASCs in Greece are on a waitlist for accommodation because of a lack of national shelter capacity [4]. Many reside in closed reception facilities or police cells and may be housed alongside adults [4], in formal or informal sites, or in street encampments, where they are exposed to a range of protection risks. Under these circumstances, sexual exploitation of UASCs is increasingly reported and visible in public parks, squares, and bars in Athens, where teenaged boys are sexually abused by older men in exchange for payment [5–9].

Sexual exploitation of children is illegal in Greece. Greece is a party to the Optional Protocol to the Convention on the Rights of the Child on the Sale of Children, Child Prostitution and Child Pornography, which requires member states to criminalize transactional sex with children [10]. Sexual exploitation is also both a human rights violation and an urgent public health concern. This form of harm is associated with a range of negative health consequences among male and female survivors, including HIV and other sexually transmitted infections (STIs), post-traumatic stress disorder (PTSD), depression, substance use, cutting behaviors, and suicidal ideation [11–13]. In humanitarian settings, where refugees and migrants often face increased barriers to sexual and reproductive healthcare and social support [14], prevention is even more urgent.

Although adolescent boys comprise a substantial majority of the population of UASCs, they are rarely the focus of policy discussions and humanitarian response efforts. Measures to address the specific health and protection needs of males are consistently left out of gender-based violence programming. Very little is known about sexual exploitation among boys in particular; the majority of research and interventions have targeted women and girls [15]. Further, traditional child protection approaches may not be appropriate to the needs of UASCs, most of whom are older adolescent boys who have migrated independently across countries and enjoy significant autonomy. In the midst of the crisis, practitioners have struggled to rapidly develop interventions with very little evidence-based programming or guidance. This paper addresses the state of the evidence regarding the sexual exploitation of UASCs and explores 3 potential approaches to prevention in Greece.

## State of the evidence

Research on the risks and experiences of sexual exploitation among male and female UASCs, both within Greece and globally, is extremely limited, and little is known about their specific vulnerabilities. The “Safer UK” study (2007), the primary study on the topic, identified several factors contributing to UASCs’ vulnerability to exploitation [16]. Factors included limited understanding of their legal rights, mixed-age or mixed-sex accommodation, a lack of supervision, isolation from social networks and family members, and a lack of confidence in social workers’ responses to calls for help [16]. A smaller-scale study of UASCs in London highlighted that a combination of unmet emotional needs, material insecurities, fear of forced removal, and limited social welfare support makes unaccompanied youth increasingly dependent on others and vulnerable to exploitative relationships [17]. Three other studies involving UASCs describe sexual exploitation and abuse by traffickers and agents during migration as a condition of, or to accelerate, their movement or to repay debts [18–20]. A recent UNICEF study also noted the gendered aspects of sexual exploitation, highlighting that although young women are more likely to be targeted, Afghan boys are particularly vulnerable because of the greater social acceptance of the sexual exploitation of young boys rather than girls in some areas of Afghanistan [19].

Recent literature on trafficking and exploitation in humanitarian settings more broadly highlights that displacement settings can exacerbate and create new risks associated with sexual exploitation. Risk factors include the erosion of the rule of law, lack of legal status, restrictions on movement [21], and the development of criminal networks to target new victims such as refugees [21,22]. A lack of employment opportunities, inadequate access to protection and support services [22,23], insufficient and inequitable distribution of food and aid [23, 24], and a loss of family and community support mechanisms also increase vulnerability to exploitation and abuse [22,24].

International guidance addressing child protection, trafficking, and exploitation does not address the intersection of sexual exploitation and UASCs. Further, evaluations of targeted prevention interventions and programing are absent from the literature.

## Emerging approaches to prevention

In Greece, 3 approaches to prevention have been heavily debated as strategies for reducing vulnerability to sexual exploitation among UASCs: high-security shelter, life skills education, and cash transfer programming. In light of these policy discussions and the urgent need for guidance, the following section explores the available evidence.

### Shelter models: Prevention through protection

The urgent need to accommodate the influx of UASCs in Europe, along with concerns about risks of sexual exploitation, has raised questions about the most appropriate model of shelter for them. Practitioners in Greece and beyond are debating the prevention potential of protection measures such as enhanced security, supervision, and movement restriction.

In both humanitarian and general settings, research on gender-based violence shelter programs is largely focused on programs serving survivors of domestic violence or gender-based violence in general. It does not address the specific vulnerabilities and protection concerns of survivors of sexual exploitation and the related harm of trafficking, such as the complex relationship between survivors and exploiters [25–29]. Similarly, most literature addressing shelter provision for sexually exploited youth focuses on programs serving girls in the United States [30–33].

There is a lack of consensus regarding how to balance the security and protection of survivors of sexual exploitation and trafficking with their rights to personal mobility [34]. On one hand, survivors’ lack of legal status [35], likelihood of running away [33,35], participation in prosecution and investigation processes [35], and need for physical protection from traffickers [30,35] are cited as key reasons for restricting UASCs’ ability to freely enter and exit shelters. On the other hand, researchers highlight that overly restrictive containment of children borders on detention, potentially violating children’s human rights. They emphasize that decisions regarding the placement of the child should be made on a case-by-case basis, in the best interests of the child [35]. Service providers and advocates also assert that lockdown programs reinforce a sense of loss of control and can inhibit the healing process [33]. Research on gender-based violence shelters in humanitarian settings highlights that severe restriction on mobility can also create a feeling of being imprisoned and deter survivors from seeking shelter in the first place [36].

Evaluations of shelter programs serving unaccompanied adolescent boys at risk of sexual exploitation are needed to inform program design that both facilitates healing and prevents further exploitation.

### Life skills: Youth empowerment and healthy decision-making

Alongside protection, UASCs also need the knowledge and skills to support healthy decision-making and coping strategies. A recent study involving focus groups with 120 refugee and migrant children in Greece highlighted that stress and uncertainty about their situation were linked to a loss of hope and engagement in negative coping behaviors [37]. Finding that youth had poor access to education, skill-building opportunities, healthcare, and protective environments, the study authors recommended life skills training to address these challenges [37].

The World Health Organization (WHO) has identified 5 life skills to be relevant across cultures: (1) decision-making and problem-solving, (2) creative thinking and critical thinking, (3) communication and interpersonal skills, (4) self-awareness and empathy, and (5) coping with emotions and coping with stress [38]. Life skills training curricula have been delivered to refugee and migrant youth in various contexts, including Lebanon, Jordan, Syria, Palestine, Burundi, and Ivory Coast [39–42]. Although life skills training approaches are not new, research and evaluation of programs in refugee and migrant settings are scarce.

Life skills education may be a promising approach to the prevention of sexual exploitation of UASCs, as it treats youth as having agency to recognize and navigate risks in their environments. However, a review of life skills training programs in 40 countries by UNICEF in 2012 found that less than half of the curricula addressed sexual abuse and exploitation [42]. A targeted life skills curriculum to promote sexual health and prevent sexual exploitation among refugee and migrant boys has recently been developed in Greece. Evaluation of this pilot program and others is urgently needed.

### Cash transfers: Addressing economic vulnerability

Cash transfer programming, the direct distribution of money to individuals, is increasingly implemented in crisis settings and may be a promising strategy to reducing economic vulnerability associated with sexual exploitation. However, the inclusion of UASCs as direct recipients in large-scale cash transfer programming has been heavily debated among practitioners in Greece. Concerns regarding risks of harm, optimal transfer amount and age criteria, and conditionality with education or other programming require further research.

Lessons may be drawn from the literature addressing cash transfer programming targeting minors and the impact of cash transfer programming on sexual exploitation more broadly.

Evidence of programs specifically targeting UASCs was not identified in the literature. However, 4 programs directly targeting minors as cash transfer recipients in the US, Malawi, and South Africa did not indicate significant harmful effects [43–46]. Three of the 4 programs assessed risks of harm and found that cash transfers were not used to purchase drugs or alcohol or finance other illicit behaviors by the vast majority of participants [44–46]; in fact, 2 programs observed a decrease in substance use among enrolled adolescents [45,46]. In South Africa, social harm was reported by only 16 out of 2,448 participants of a cash transfer program, the majority of incidents involving minor teasing for participation in the study [44]. Evidence from these programs suggests that providing cash transfers directly to youth may be a safe and viable option.

The impact of cash transfer programming on sexual exploitation among minors is varied. While some studies found that cash transfers reduced the exchange of sex for basic survival needs such as cash and food among young women [47–49], others did not demonstrate a significant effect [44,50]. Stronger evidence supports the positive impact of cash transfers on adolescent sexual health and behavior, including reductions in rates of HIV and herpes simplex virus 2 (HSV-2) [43], HIV-risk behavior [48], and the likelihood of reporting unprotected sex [44]. Programs combining cash transfers with life skills education and support services have demonstrated particularly strong results [45,48,51].

Although evidence from other contexts points to the potential of cash transfers to address sexual exploitation among youth generally, cash transfers targeting UASCs have yet to be evaluated.

## Conclusion

Preventing sexual exploitation and other forms of gender-based violence requires an ecological approach in which risk factors at multiple levels, including the structural, community, family, and individual levels, are addressed [52]. Shelters may offer an alternative to traditional family and community supports in displacement settings. Life skills approaches aim to empower individuals with the knowledge and skills required to make healthy choices and navigate risks in their environments. Cash transfer programming aims to improve UASCs’ positions of economic vulnerability. A combination of these approaches, as well as others such as guardianship, mentoring programs, supportive identification and referral procedures by law enforcement, and demand-side strategies to strengthen the arrest and prosecution of perpetrators, is likely required in order to significantly reduce vulnerability to sexual exploitation among UASCs [53].

Unaccompanied refugee and migrant children, the majority of whom are adolescent boys, are particularly vulnerable to exploitation and abuse. While gender-based violence programming is justifiably focused on women and girls in light of their heightened vulnerability, the specific prevention and response needs of boys must not be overlooked. Research on the gendered risks, experiences, and needs of boys related to sexual exploitation is urgently needed to inform the design of responsive policies and programs.

With the rising number of unaccompanied refugee and migrant boys on the move, rigorous evaluation of piloted interventions will be essential to developing the guidance, tools, and other resources so urgently needed by practitioners.

## Abbreviations

HSV-2: herpes simplex virus 2
PTSD: post-traumatic stress disorder
STI: sexually transmitted infection
UASC: unaccompanied and separated child
WHO: World Health Organization
